# BODIPY-Based Molecules for Biomedical Applications

**DOI:** 10.3390/biom13121723

**Published:** 2023-11-30

**Authors:** Sarasija Das, Sudipto Dey, Sanujit Patra, Arindam Bera, Totan Ghosh, Bibin Prasad, Kapil Dev Sayala, Krishnendu Maji, Anjan Bedi, Sashi Debnath

**Affiliations:** 1 Department of Chemistry and Biochemistry, The Ohio State University, Columbus, OH 43210, USA; das.448@osu.edu; 2 Department of Chemistry, Jadavpur University, Jadavpur, Kolkata 700032, India; sudiptodey608@gmail.com; 3 Department of Applied Chemistry, Maulana Abul Kalam Azad University of Technology, Nadia 741249, India; sanujitpatra692@gmail.com (S.P.); arindambera.arindam2018@gmail.com (A.B.); totan.chem@gmail.com (T.G.); 4 Solenic Medical, Inc., 4275 Kellway Circle, Suite 146, Addison, TX 75001, USA; bibin.prasad@solenic.com; 5 Department of Chemistry, Southern Methodist University, 3215 Daniel Avenue, Dallas, TX 75206, USA; ksayala@smu.edu; 6 Department of Radiology, University of Texas Southwestern Medical Center, Dallas, TX 75390, USA

**Keywords:** BODIPY, BODIPY dyes, fluorescent probe, BODIPY-based functional materials for medical use

## Abstract

BODIPY (4,4-difluoro-4-bora-3a,4a-diaza-s-indacene) derivatives have attracted attention as probes in applications like imaging and sensing due to their unique properties like (1) strong absorption and emission in the visible and near-infrared regions of the electromagnetic spectrum, (2) strong fluorescence and (3) supreme photostability. They have also been employed in areas like photodynamic therapy. Over the last decade, BODIPY-based molecules have even emerged as candidates for cancer treatments. Cancer remains a significant health issue world-wide, necessitating a continuing search for novel therapeutic options. BODIPY is a flexible fluorophore with distinct photophysical characteristics and is a fascinating drug development platform. This review provides a comprehensive overview of the most recent breakthroughs in BODIPY-based small molecules for cancer or disease detection and therapy, including their functional potential.

## 1. Introduction

**Figure 1:**
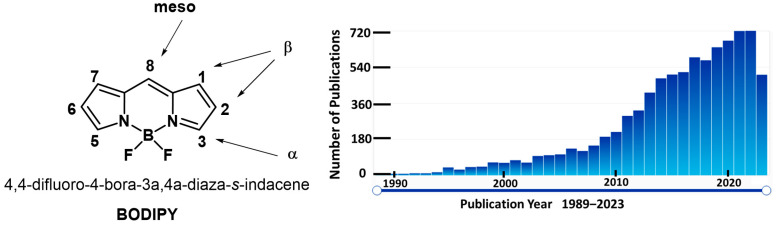
Core structure of 4,4-difluoro-4-bora-3a,4a-diaza-s-indacene (BODIPY, **left**) and references searched with the phrase “BODIPY” in scifinder-n.cas.org (**right**).

In 1968, the first BODIPY was synthesized by Trelibs and Kreuzer [1]. BODIPY derivatives have attracted much attention as fluorescent probes in various applications due to their unique properties [2,3]. BODIPY/boron dipyrromethene/4,4-difluoro-4-bora-3a,4a-diaza-s-indacene is a class of organic dyes that exhibits strong absorption and emission in the visible and near-infrared regions of the electromagnetic spectrum (Figure 1) [4]. These dyes are extremely fluorescent and have excellent photostability, which make them perfect for imaging and sensing applications. The tunable [5] fluorescence capabilities of BODIPY derivatives are one of the key factors that have drawn significant interest [6]. Changing the structure of the BODIPY core and adding various functional groups can fine-tune the absorption and emission wavelengths of these dyes. This tunability allows for the design of BODIPY derivatives with specific fluorescence properties suitable for different applications [7].

Cancer is a challenging condition that involves uncontrolled cell division and proliferation [8]. Because of its complex nature, the tendency to metastasize, and its resistance to standard treatment, it poses significant challenges in medicine and healthcare [9]. Considerable efforts have been made to create successful cancer therapies, such as surgery, chemotherapy, radiation therapy, immunotherapy, photodynamic therapy, photothermal therapy and targeted therapies [10,11]. Despite these advances, certain types of cancer remain challenging to treat [12], and the side effects of present treatments can be severe [13]. Recently, researchers started looking into innovative approaches to address the challenges posed by cancer and improve therapeutic outcomes [14]. One such promising method is to use BODIPY as a versatile pharmacological platform [15].

BODIPY derivatives have been used not only in biological applications but also in other fields [16]. Due to their high fluorescence quantum yields and exceptional thermal and chemical durability, they have been used as fluorescent dyes in optoelectronic devices, including organic light-emitting diodes (OLEDs) [17] and organic photovoltaics (OPVs) [18]. Additionally, BODIPY-based sensors have also been utilized for environmental monitoring, the detection of pollutants, and food quality control [19]. Derivatives of BODIPY are frequently used in biological imaging [10]. They are excellent for observing cellular structures and processes because of their fluorescent characteristics. They can be used to identify certain biomolecules, like proteins or nucleic acids, and monitor their dynamics and localization inside cells [20]. BODIPY derivatives can also be used as sensors for different analytes, including ions, pH, reactive oxygen species, and enzymatic activities, providing real-time monitoring of biochemical processes in living systems [21]. Researchers have investigated BODIPY-based molecules for imaging and diagnostic uses, but their usage as pharmaceuticals for cancer treatment is still in its infancy [22]. BODIPY-based chemicals in photodynamic therapy (PDT) for cancer have shown potential improvement. Reactive oxygen species (ROS), capable of specifically killing cancer cells, are created by activating light-sensitive substances/photosensitizers in PDT [23]. BODIPY dyes have potent photostability and their beneficial photophysical characteristics render them effective photosensitizers in PDT [24]. Researchers have developed substances based on BODIPY that accumulate specifically in cancer cells and cause lethal effects when triggered by light [25]. These substances can be coupled with molecules that target cancer cells to increase their selectivity towards those cells while minimizing damage to healthy tissues [26,27]. Additionally, BODIPY dyes can be engineered to emit fluorescence, allowing for real-time monitoring of their distribution and therapeutic effects [28]. Overall, because of their wide range of uses, adjustable fluorescence characteristics, and photostability, BODIPY derivatives have gained a lot of attention as fluorescent probes.

New BODIPY compounds with improved characteristics and innovative functionalities are still being investigated in ongoing research for a variety of scientific and technical improvements. This review seeks to provide valuable insights into the potential of BODIPY as a diverse and influential platform for solving cancer treatment challenges by critically analyzing the present state of research. The review’s findings may pave the way for developing novel and targeted cancer medicines, bringing us closer to better treatment outcomes and quality of life for cancer patients.

## 2. Properties of BODIPY-Based Compounds

### 2.1. BODIPY-Based Fluorescent Compounds

Due to their outstanding fluorescence characteristics, BODIPY dyes are widely employed in fluorescence applications [16,28]. Depending on the molecular structure, BODIPY dyes can absorb light in the ultraviolet (UV) or visible range [29]. The absorption maximum is usually between 400 and 600 nm. BODIPY dye emission maxima are often redshifted from absorption maxima, falling between 500 and 700 nm [30]. This substantial shift reduces self-absorption and enables the efficient detection of emitted fluorescence. BODIPY dyes are well-known for their high fluorescence quantum yields, typically near 0.8. Quantum yield, defined as the ratio of released photons to absorbed photons, reflects the efficiency of fluorescence emission [31]. The high quantum yields of BODIPY dyes suggest that a considerable percentage of the absorbed energy is transformed into fluorescence. The combination of high quantum yield and photostability contributes to BODIPY compounds overall brightness [29]. The brightness of BODIPY dyes makes them useful for applications needing strong and long-lasting fluorescence signals. Some BODIPY derivatives exhibit solvent sensitivity, meaning their fluorescence properties are affected by their surroundings, particularly the polarity of the solvent [32]. This feature can be used to create BODIPY dyes that operate as polarity-sensitive probes or indicators. These dyes vary their fluorescence intensity or emission wavelength in response to pH changes. pH sensitive BODIPY dyes are used in pH imaging and the monitoring of pH changes in biological systems [33].

BODIPY 493/503, a green-fluorescent BODIPY dye has been reported for the utilization of lipid droplet staining in live cells [34]. The wavelengths of light (in nanometers) at which these dyes absorb and emit fluorescence are denoted by the numbers 493 and 503. The selectivity of BODIPY 493/503 has been conceptualized with the staining of neutral lipid droplets in BHK cells and exhibits bright green fluorescence upon binding to lipid droplets. BODIPY FL is one of the unique BODIPY dyes which produces light in the green-yellow range [35]. It is frequently employed as a multipurpose fluorescent dye for various chemical and biological applications. The detection of specific DNA or RNA units based on the quenching of BODIPY FL fluorescence was established due to the interaction of BODIPY FL with a uniquely situated guanine. This finding increased the utilization of the oligonucleotide containing a BODIPY FL-modified cytosine at the 5′-end. After interacting with a target DNA, its fluorescence was quenched by the guanine in the target and the rate of fluorescence quenching was proportional to the targeted DNA quantity. Recently, it was shown that a BODIPY FL-labeled monoterpenoid (BODIPYmyrt) could exhibit a high quantum yield (~100%) [36]. BODIPYmyrt can be used to analyze the characteristics of a wide range of bacteria and pathogenic fungi since it successfully permeates the membranes of bacterial and fungal cells. BODIPY TMR emits in the red region of the spectrum and has been applied for various applications, including cell labeling, fluorescence microscopy, and flow cytometry [37]. BODIPY TMR containing exendin-4-like neopeptide conjugate was developed for the fast purification and segregation of mouse pancreatic β-cells. BODIPY TMR conjugate was utilized to target the glucagon-like peptide-1 receptor and β-cells that were >99% insulin positive could be promptly separated [37].

BODIPY 558/568 emits in the orange-red region of the spectrum. The wavelengths of light (in nanometers) at which these dyes absorb and emit fluorescence are denoted by the numbers 558 and 568. In a variety of biological and chemical research, the BODIPY 558/568 conjugates were implemented as a fluorescent label or marker [38]. These dyes are used by researchers for a variety of fluorescence microscopy techniques, cell labeling, following molecules throughout cellular processes and studying protein–protein interactions [39,40]. BODIPY 630/650, after conjugating with adenosine receptor ligand N-ethylcarboxamido-adenosine (NECA) showed the greatest potency (Log IC50 (Gi) = −9.31) at the human adenosine A1-receptor [41]. The conjugate confirmed the selective labeling efficacy of human adenosine A1-receptor in single living cells. The conjugation of BODIPY 630/650 with antagonist VUF13816 (orthosteric targeting moiety) with a peptide linker showed high binding affinity to histamine H_1_ receptor (H1R) and empowered H1R visualization by confocal microscopy [42]. Reports have shown that BODIPY TR methyl ester dye served as a useful counterstain for observing the cellular processes of morphogenesis in zebrafish embryos that were transgenic for the green fluorescent protein (GFP) [43]. This synthetic BODIPY TR conjugate showed distinct fluorescence compared to GFP emission, making it simple to capture dual-channel, three-dimensional (3D), and four-dimensional (4D) confocal imaging data sets of living organisms [43].

**Figure 2:**
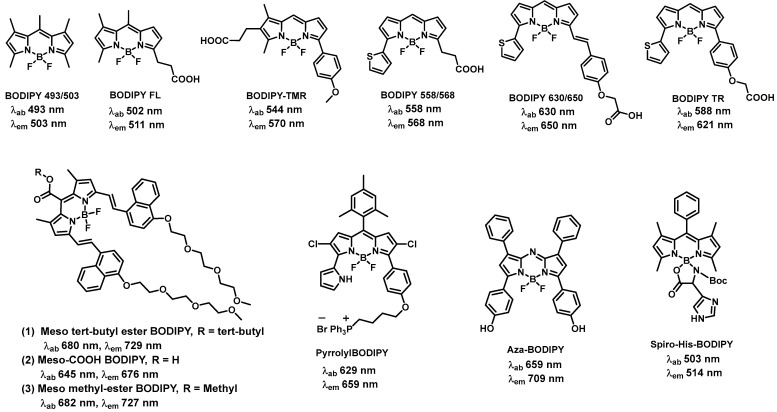
Longer-wavelength absorbing/emitting BODIPY-based fluorophores.

In the last few decades, many far-red and NIR BODIPYs have been synthesized through functionalization at the α, β, and meso sites of the BODIPY core. Wu and co-workers studied a series of meso-ester/acid-substituted BODIPY dyes functionalized with oligo(ethylene glycol) ether styryl or naphthalene vinylene groups at the α positions (a) meso tert-butyl ester BODIPY, (b) meso-COOH BODIPY, (c) meso methyl-ester BODIPY (Figure 2). The resulting derivatives become water soluble, membrane-permeable, and their absorptions and emissions are located in the far-red or near-infrared region, thereby being suitable for bioimaging applications in living cells [44]. Miao et al. developed various red to near-infrared emitting pyrrolyl BODIPY dyes and demonstrated good lipophilic character, excellent photostability, low cytotoxicity and intense NIR fluorescence of fluorophore pyrrolylBODIPY (Figure 2) in the mitochondrial of HeLa cells [45]. Rivero and co-workers synthesized, characterized, and evaluated different aza-BODIPY compounds as metal sensors and cell staining probes and they found aza-BODIPY (Figure 2) allowed them to characterize a lipotoxic condition mediated by saturated fatty acids, a critical phenomenon on β-cell damage associated with diabetes mellitus type II [46]. Wang et al. synthesized and studied a series of mono-spiro- and di-amino acid−BODIPY derivatives, containing basic (histidine, lysine, and arginine), acidic (aspartic acid), polar (tyrosine, serine, and glutamine), and nonpolar (methionine) amino acid residues for potential therapeutic and/or imaging applications [47]. The spiro-His-BODIPY (Figure 2) showed maximum internalization, favorable localization in the cell lysosomes, endoplasmic reticulum, and mitochondria.

### 2.2. BODIPY Probes Capable of Detecting and Tracking Amyloid-β Aggregates and Structural Changes

**Figure 3:**
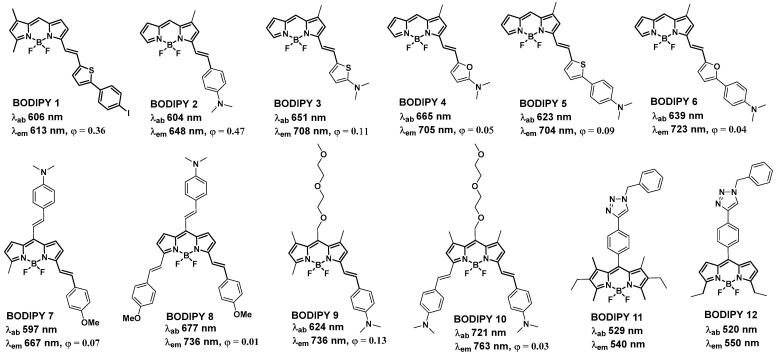
BODIPY-based probes for monitoring aggregation and conformational changes of amyloid-β.

Numerous neurodegenerative disorders, including Alzheimer’s disease, are significantly influenced by amyloid accumulation. One of the primary markers linked to this condition is the amyloid-β peptide (Aβ). Aβ aggregates have a wide variety of shapes and different pathogenic behaviors. Small molecule-based probes and sensors that can detect Aβ aggregates should improve the understanding of the processes that lead to amyloid formation and make it easier to create treatment plans that counteract amyloid neurotoxicity. Additionally, recent investigations revealed the involvement of amyloid Beta oligomer with cancer cell growth [48]. Pavlieukeviene et al., reported the inhibition of human cancer cell growth by amyloid beta oligomers [48]. The most adaptable small molecule fluorophores are BODIPY dyes. BODIPY dyes could be seen as distinctive platforms for developing sensors and probes to identify and monitor structural changes in Aβ aggregates (Figure 3).

#### BODIPY Probes for Monitoring Aggregation and Conformational Changes of Amyloids

Recently, elevated levels of Aβ amyloids in the plasma of cancer patients have been observed [49]. Qin et al. reported that Aβ amyloids can eliminate the cancer stem cell through iron-mediated ROS production [50]. Many studies have investigated the interactions between BODIPY dyes and Aβ aggregates. The following requirements should be met by a viable probe in order to detect Aβ aggregates from AD homogenates: (a) the ability to cross the blood–brain barrier (BBB) [51], (b) near-IR emission, ideally over 650 nm; (c) unique marking of the Aβ aggregates, with fast clearance of the free molecules; and (d) a change in the photophysical features of the probe upon binding with Aβ aggregates [52,53]. The BODIPY 1 [54] was initially developed to serve as a nuclear and fluorescent imaging probe for the in vitro imaging of amyloid aggregates found in AD brains. With respect to Aβ aggregates, BODIPIY 1 demonstrated a decent in vitro binding affinity (in the 100 nM range) and approached the properties of a functional Aβ imaging probe due to its emission at 615 nm. More recently, investigation into the development of efficient probes revealed that the diethylamino styryl group containing probe BODIPY 2 [55] (BAP-1, Figure 3) showed increased in vitro binding affinity to Aβ aggregates (K_d_ = 44 nM), and showed a redshifted emission maximum closer to the desired near-IR range (λem ≈ 650 nm). The diethylamino styryl group is crucial for binding to Aβ aggregates. BODIPY 2 is a member of a group of dyes known as molecular rotors [56] in which the BODIPY unit serves as the acceptor and dimethyl aniline as the donor.

Further substitutions of the dimethylaminophenyl group in BODIPY 2 with a dimethylaminothiophenyl group (BODIPY 3), a dimethylaminofuranyl group (BODIPY 4), a dimethylaminophenylthiophenyl group (BODIPY 5), and a dimethylaminophenylfuranyl group (BODIPY 6) exhibited Aβ-binding affinities in the 20–150 nM range [57]. For BODIPYs 3–6, the emission was further redshifted to the 700 nm range, which could be beneficial for in vivo imaging experiments. Surprisingly, in the presence of equimolar quantities of Aβ1-42 fibrils, these probes dramatically increased emission intensities and the highest increase of 23-fold was observed for BODIPY 6. These probes demonstrated extremely high specificity for amyloid aggregates because of the emission amplification in the presence of substantial excesses of bovine serum albumin (BSA; 680 µM). Attempts have been made to extend the conjugation of the BODIPY core by substituting in both the 3 and 5 positions along with the meso position to produce BODIPY 7, BODIPY 8, BODIPY 9, BODIPY 10 [53]. The extended conjugation keeps the emission maxima of these molecules in the 650–760 nm range. BODIPY 8, with a considerably higher K_d_ value (320 nM), showed no discernible staining patterns in Tg2576 mouse brain sections. On the other hand, probes BODIPY 7 and BODIPY 9 showed effective staining in the brain sections and displayed good patterns, while they exhibited altered K_d_ values (230 nM and 50 nM, respectively). Although the K_d_ value for BODIPY 10 was relatively low (97 nM), the staining was not as strong as for BODIPY 7 and BODIPY 9. The brain region of interest was best stained by the BODIPY 9 with the lowest K_d_ value.

The addition of a triazole moiety to the meso position of BODIPY dyes resulted in detectable fluorescence amplification in the presence of soluble oligomeric Aβ1-42 constituents. As a result, BODIPY 11 and BODIPY 12 detected a conformational change from unordered to ordered Aβ1-42 oligomers [52]. BODIPY 11 and BODIPY 12 were able to perceive prefibrillar soluble aggregates as a continuous increase in fluorescent intensity demonstrated by monitoring the time-dependent aggregation of Aβ1-42 oligomers [58]. The binding studies agreed with the circular dichroism (CD) and light scattering data, which were utilized to track the conformational changes of Aβ1-42 oligomers [59]. Additionally, significant magnitudes of emission intensity were obtained in the presence of an excessive amount of Aβ1-42 with respect to the concentrations of BODIPY 11 and BODIPY 12, and the kinetics of the Aβ aggregation mechanism were not influenced. BODIPY 11 was employed in a dye-binding test to assess the impact of multiple peptide inhibitors of Aβ1-42 aggregation [60]. Furthermore, the BODIPY 11 assay was compared with a ThT-based assay, illustrating both dyes’ complimentary behavior and the importance of multi-sensor techniques for tackling complicated amyloid-related processes [59,60]. ThT, as a fibril-specific sensor, could not identify oligomeric Aβ1-42 species present during the initial phases of Aβ aggregation. Thus, ThT might be poorly suited to evaluating inhibitors during the preliminary phases of amyloid oligomerization. On the other hand, a steady rise in BODIPY 11 intensity was observed over 15 h of Aβ oligomer aggregation, implying that the sensor was able to identify early-stage Aβ aggregates [60].

### 2.3. Detection of Reactive Oxygen Species

**Figure 4:**
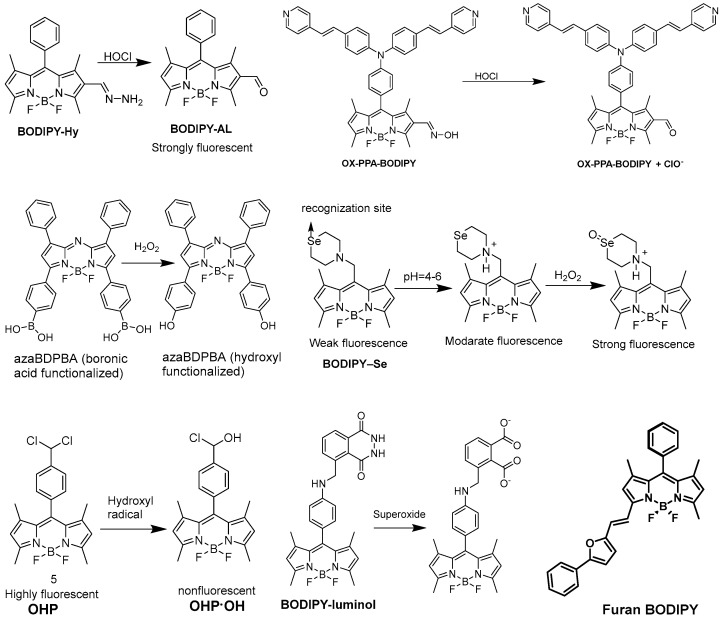
BODIPY-based probes for the detection of reactive oxygen species.

Generally, in living organisms, various types of reactive oxygen species (ROS) are formed which are chemically reactive, playing an important role in biological processes. This ROS plays a significant role in signaling and pathological states, but the overproduction of ROS shows some oxidative stress. Here, we showed some BODIPY-based fluorescent probes for this application (Figure 4). Hypochlorite (ClO^−^), a renowned ROS is generated from the reaction of H_2_O_2_ and chloride ions in the presence of myeloperoxidase (MPO). ClO^−^ in equilibration with hypochlorous acid (HOCl) fundamentally plays an important role for the immune system but overproduction of HOCl/ClO^−^ causes various types of diseases. In 2015, Zhou et al. [61] reported a fluorescent-based “turn on” probe BODIPY-Hy (non-fluorescent) for the determination of HOCl (Figure 4). Without HOCl, it was weakly emissive due to the isomerization of the C=N bond. By treating with HOCl, it generated a sudden increase of more than 11 times in emission intensity due to the oxidation of the C=N bond to produce the compound BODIPY-AL (strongly fluorescent). BODIPY-Hy is generally used for the imaging of intracellular HOCl, having low cytotoxicity. By leveraging a similar idea, Qian et al. [62] also developed a weak fluorescent probe OX-PPA-BODIPY (turn-off), in which an aldoxime group is at position two of the BODIPY skeleton (Figure 4). Due to this type of C=N isomerization, it showed a weak fluorescence but, in the presence of HOCl, the fluorescent emission was exceptionally increased (turned on in OX-PPA-BODIPY + ClO^−^) more than 20 times and switched the color from pink to green.

Hydrogen peroxide H_2_O_2_ plays an important role in the regulation of immune responses, but excessive production of H_2_O_2_ results in numerous diseases. The identification of H_2_O_2_ by applying fluorescent probes has drawn a large amount of interest. Jiang et al. presented a BODIPY-based NIR fluorescent probe azaBDPBA (boronic acid functionalized) for the detection of H_2_O_2_ (Figure 4) [63]. It showed a long absorption/emission wavelength in the NIR area, remarkable photostability and high selectivity towards H_2_O_2_ over other ROS. Qian et al. reported a lysosome-targeted fluorescent probe BODIPY–Se, which enabled photoinduced electron transfer (PET) for the detection of H_2_O_2_ (Figure 4) [64]. It is composed of a selenamorpholine group and a BODIPY core. The protonation of nitrogen atoms in selenamorpholine modifies the electronic state, causing the PET process to be quenched and fluorescence to be recovered. The fluorescence intensity of BODIPY–Se rose progressively as the pH was reduced from 7 to 3. Guo et al. [65] derived a near infrared fluorescent probe which consisted of aza-BODIPY fluorophore, hydrophilic polyethylene glycol, a biotin segment as a targeting group and pinacol borate as a recognition unit for the in vivo sensing of H_2_O_2_. It was designed using the common oxidative cleavage of the boronate esters approach and ITC process. Hydroxyl radicals [66]/organic radicals [67] have biological importance as they are highly effective fluorescent indicators. So, despite being some of the most active species, they can react with biomolecules and thus cause harm to the cell. Alsaedi et al. [66] designed a BODIPY-based OHP probe, in 2017, for the sensitive determination of endogenous hydroxyl radicals. When hydroxyl radicals were added to the OHP solution, the emission peak at 514 nm was quenched due to the formation of a non-fluorescent product with one chlorine atom substituted by hydroxyl. Superoxide is an additional important ROS that is required for human survival, notably in cell development and metabolism. Rochford et al. developed a benzyl linked BODIPY-luminol conjugate [68], in 2015, using chemiluminescence resonance energy transfer (CRET) to detect superoxide. Here, luminol chemiluminescent was used as donor, BODIPY as an acceptor. The CRET between the luminol and the BODIPY was suppressed under normal physiological conditions. When H_2_O_2_ and CuSO_4_ were incorporated into the BODIPY-luminol solution, double chemiluminescence, one from immediate luminol and another from CRET emission, was observed. In 2018, Kayal et al. reported the imaging of intracellular singlet oxygen with phenylfurylethenyl-substituted BODIPY (Figure 4) [69]. The cycloaddition of singlet oxygen to the furan rings significantly affects the electronic environment of the BODIPY core through the disruption of the furan ring.

### 2.4. BODIPY-Based Probes in Cancer Detection

**Figure 5:**
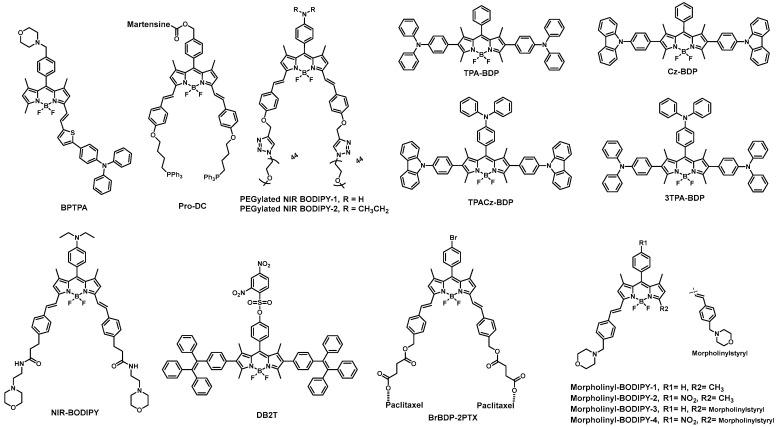
BODIPY-based probes for cancer detection.

Cancer is the second largest contributor to mortality worldwide and is caused by the uncontrolled growth and division of cells. Despite significant efforts to cure cancer, the success of these therapeutic techniques remains a challenge. Early and precise cancer detection may be a better method to increase cancer survival rates than improving cancer therapy. Fluorescent probe-based fluorescence imaging is widely used for cancer imaging and has tremendous importance in cancer diagnosis due to its distinct benefits over traditional imaging approaches. Chen et al. developed a novel compound, BPTPA, which is a triphenylamine-conjugated BODIPY molecule (Figure 5) [70]. The researchers investigated the binding affinity of BPTPA with G4 DNA structures and found that it selectively binds with G3T3 G4 DNA, leading to the formation of a water-compatible nanocomplex, BPTPA-G3T3. The BPTPA-G3T3 complex was found to have the ability to image mitochondria and inhibit the expression of TrxR2. In addition, BPTPA-G3T3 can reduce the membrane potential of mitochondria and impede the proliferation of BGC-823 cancer cells, making it a promising candidate for cancer imaging and chemotherapy. Das et al. developed a new prodrug conjugate Pro-DC [71] (Figure 5) that is specific to mitochondria. Upon irradiation with a 400 nm source at 1.0 mW cm^2^, the prodrug undergoes a photocleavage reaction, releasing mertansine and CO, along with a BODIPY derivative as a luminescent marker with an emission peak at 655 nm, in the mitochondrial matrix. The effectiveness of the prodrug conjugate Pro-DC was demonstrated using MCF-7 cells, and the intracellular luminescence could be effectively visualized. The results suggest that this prodrug conjugate can be used as an image-guided combination therapy for the mainstream treatment of cancer. Overall, the study offers a promising strategy for targeted drug delivery and imaging in cancer therapy.

Siegwart et al. utilized the photoinduced electron transfer (PET) effect to develop two water-soluble, pH-adjustable near-infrared (NIR) BODIPY fluorescent probes, PEGylated NIR BODIPY-1 and PEGylated NIR BODIPY-2 (Figure 5) [72]. These probes exhibit near-infrared (NIR) emissions within the range of 650–900 nm. This probe is capable of localizing in cancer cells within the body and is activated by acidic pH, leading to high signal contrast between mice with lung or cervical cancer and normal mice. Moreover, probe PEGylated NIR BODIPY-2 can locate tumors in mice and become activated after intravenous injection. In mice carrying MDA-MB-231 subcutaneous tumors, strong and continuous radiation was observed upon injection of either probe PEGylated NIR BODIPY-2 or probe PEGylated NIR BODIPY-1. Overall, these findings suggest that the pH-responsive probe developed in this study has great potential for in vivo tumor imaging. Tang et al. developed a novel method to synthesize a new type of red luminescent compounds with aggregation-induced emission (AIE) characteristics by attaching triphenylamine units to the BODIPY core (TPA-BDP, Cz-BDP, TPACz-BDP, and 3TPA-BDP, Figure 5) [73]. They further fabricated corresponding functionalized AIE nanoparticles (NPs) in the presence of DSPE-PEG2000. Three AIE-active red-emissive BODIPY derivatives (TPA-BDP, Cz-BDP, TPACz-BDP, and 3TPA-BDP) were rationally designed and synthesized in this study. Three NPs based on these AIE luminogens (AIEgen) exhibited bright red photoluminescence with high fluorescence quantum yield in aqueous media. These NPs were uniformly dispersed in water and demonstrated excellent stability and good biocompatibility. They were readily internalized by HeLa cells and successfully traced for over 15 days in living cells and mice. Overall, this study offers a promising approach for the development of AIE-active red-emissive NPs with excellent biocompatibility and stability [74] which could potentially be used for long-term imaging in living systems. Ting et al. developed a fluorescent NIR-BODIPY probe (Figure 5) that is activated by acidic pH and specifically targets lysosomes for in vivo imaging [75]. This probe exhibited strong adsorption and fluorescence spectra with maximum peaks at 644 nm and 658 nm, respectively. Additionally, the probe’s quantum yield increased 41.9-fold with a decrease in pH from 7.4 to 4.4. To improve water solubility, the researchers packaged NIR-BODIPY with DSPE-PEG2000 to create water-soluble NIR-BODIPY nanoparticles (NIR-BODIPY-NPs). These NIR-BODIPY-NPs were able to enter the lysosomes of living cells and were selectively delivered to tumor tissue via a passive enhanced permeability and retention (EPR) effect, enabling the visualization of solid tumors. Overall, this study presents a promising approach for the development of a lysosome-specific probe that can be used for in vivo tumor imaging.

The development of multifunctional probes that can effectively detect hydrogen sulfide while also serving as a photodynamic therapy for cancer cells is currently a major demand in the scientific community, yet still poses a significant challenge worldwide. In their research, Ye et al. have developed a BODIPY-based fluorescent probe, called probe DB2T (Figure 5), which is capable of H_2_S fluorescence “turn-on” detection and light-sensitive anticancer activity [76]. The probe exhibits high selectivity towards H_2_S, a low detection limit of 6.39 nM, and good biocompatibility. It has been successfully used for fluorescence imaging of exogenous and endogenous H2S in zebrafish, HCT116 cells, and tumor tissues. Additionally, the phototherapy effect of the probe has been evaluated and shown to be effective in the photodynamic ablation of HCT116 cancer cells under white light irradiation. The study is expected to contribute to the development of multifunctional probes for simultaneous H_2_S detection and photodynamic therapy in cancer cells. In 2018, Zheng et al. developed an amphipathic near-infrared brominated BODIPY-paclitaxel conjugate (BrBDP-2PTX) (Figure 5) to achieve both NIR fluorescence monitoring and chemical therapy for tumors [77]. This conjugate is composed of a brominated BODIPY dye (a hydrophobic moiety) and two paclitaxel molecules (PTX, a hydrophilic moiety) linked by covalent bonds. The self-assembly of BrBDP-2PTX easily formed stable nanoparticles (BrBDP-2PTX-NPs) with a diameter of 131 nm via the nanoprecipitation method. Due to aggregation-induced quenching (ACQ), the BrBDP-2PTX-NPs exhibited minimal fluorescence; nevertheless, they displayed a swift fluorescence response to proteinase K, which caused the nanoparticles to disintegrate and release the active drug PTX and the NIR BODIPY fluorophore. Confocal cellular imaging confirmed that BrBDP-2PTX-NPs were localized in lysosomes and effectively eliminated cancer cells. Upon intratumoral injection of BrBDP-2PTX-NPs, U14 tumors exhibited significant in vivo fluorescence from as early as 5 min, which remained intense even at 22 days post injection. These findings show that PTX is rapidly released in tumor areas and that nanoparticles have a long retention time frame in tumors, which improves slow drug release and long-term tumor therapy. Lu et al. developed a series of fluorescent probes called morpholinylstyryl-substituted BODIPYs (morpholinylstyryl BODIPY-1/2/3/4, Figure 5) to target lysosomes in hypoxic cells [78]. These probes were designed to incorporate a p-nitrophenyl group, which led to low fluorescence intensity due to an oxidative photoinduced electron transfer (PET) process. However, they discovered that the fluorescence intensity was fully restored upon conversion of the nitro group to the amine group, which occurred specifically under hypoxic conditions. Their investigation revealed that two of the probes, morpholinylstyryl BODIPY-1 and morpholinylstyryl BODIPY-3, had strong fluorescence and effectively targeted lysosomes in HepG2 cells. Additionally, the probes morpholinylstyryl BODIPY-2 and morpholinylstyryl BODIPY-4 demonstrated excellent lysosome-targeting capability and specificity for imaging lysosomes in hypoxic cancer cells through laser confocal imaging. These findings provide a rational design strategy for developing lysosome-targeting probes with a turn-on response in hypoxic cells, which could potentially aid in the diagnosis and treatment of cancer.

### 2.5. BODIPY Derivatives for PDT and PTT Applications

**Figure 6:**
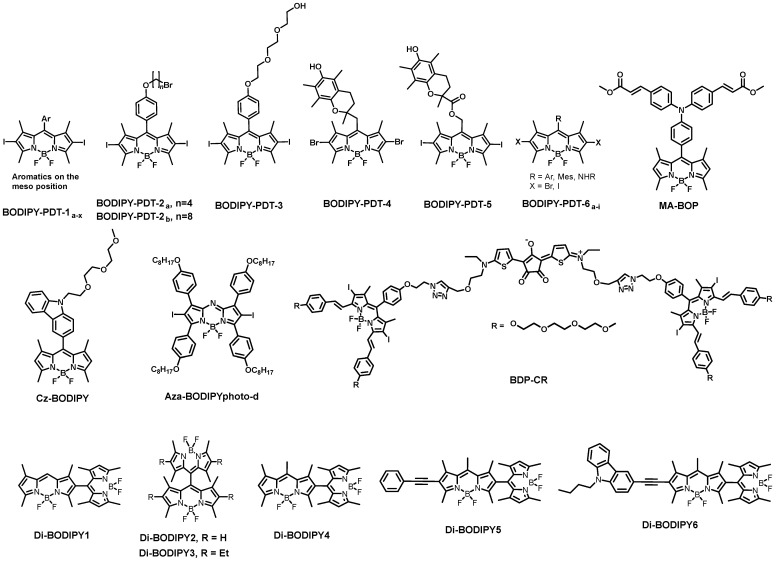
BODIPY derivatives for PDT and PTT applications.

Other than being excellent biomarkers, properly designed BODIPYs can be deployed as photosensitizers in photodynamic therapy (PDT). PDT involves light and a photosensitizing chemical compound in combination with molecular oxygen to destroy abnormal cells. Over the last decade, a novel category of PDT agents based on the BODIPY core has evolved. (Figure 6). Heavy-atom (such as halogens and metal ions) substitution in the BODIPY core is a general strategy to attribute the photosensitizing quality. The appropriate placing of heavy atoms on the BODIPY core promotes spin-orbit coupling (SOC), hence intersystem crossing (ISC), which promotes reactive oxygen species (ROS) production to destroy cancerous cells via various cell death pathways.

Caruso et al. studied the photodynamic activity of a series of BODIPYs (BODIPY-PDT-1_a-x_, BODIPY-PDT-2_a-b_) equipped with an aromatic ring in meso position and iodine atoms in β-positions [79,80]. Kim et al. performed in vitro photodynamic studies of a BODIPY-based photosensitizer BODIPY-PDT-3 which exhibited highly efficient singlet-oxygen sensitization and photo-induced necrotic cell death in Lewis lung carcinoma cells [81]. Cosa and co-workers showed ROS mediated the activation of a singlet oxygen photosensitizer and observed that the intramolecular switch introduced by the trap segment offered a better control and prevention of ^1^O_2_ sensitization in BODIPY-PDT-4 than BODIPY-PDT-5 [82]. Epelde-Elezcano et al. studied modulation of ^1^O_2_ generation in halogenated BODIPY dyes BODIPY-PDT-6_a-i_ and demonstrated how the I atom favors the ISC over the Br atom [83]. Zhao and co-workers developed a NIR monoiodinated aza-BODIPY photosensitizer with excellent in vivo efficacy for photodynamic therapy [84]. Highly efficient triplet BODIPY derivatives were developed by Yoon and co-workers for efficient photodynamic therapy and bioimaging [85]. Recently Qian et al. reported heavy-atom-free orthogonally donor–acceptor-framed BODIPY for intracellular photodynamic therapy, e.g., MA-BOP and Cz-BODIPY [86,87]. The orthogonally linked donor–acceptor-based pi-conjugating system induced an intersystem crossing. Wang et al. achieved a record low-dose photodynamic therapy (PDT)-augmented immunotherapy by developing a heavy-atom-free helical BODIPY derivative [88]. Systemic review articles are reported for heavy-atom-free BODIPY-based photosensitizers for the application of photodynamic therapy [89,90].

BODIPY has unique photophysical properties such as intense absorption in the near-infrared (NIR) region (λ_abs_ > 650 nm), NIR-I/II emissions (650–1700 nm), and tunable fluorescence quantum yields, which in turn favor NIR-I/NIR-II fluorescence imaging (FLI), photoacoustic imaging (PAI), photothermal imaging (PTI) in vivo. It has been observed that they are ideal candidates for making diverse application-oriented fluorescent probes. Additionally, BODIPY has excellent photochemical stability, low toxicity, good biocompatibility, and scope for derivatization. In particular, because of its tunable multi-imaging and multi-therapy modalities, BODIPY-based theranostics have garnered significant research interest in cancer therapy. BODIPY-based theranostics have been widely used in cancer therapy with an increasing success rate. An ideal photosensitizer for photodynamic therapy should have tumor-targeting ability, amphiphilicity for tumor cell binding, negligible dark toxicity for biocompatibility, outstanding triplet state generation efficiency to produce an ample amount of ROS, intense NIR absorption (above 650 nm) for deep penetration, and desirable characteristics of pharmacokinetics (e.g., rapid clearance from the normal tissues) [91]. Kim and co-workers reported a photoactivatable fluorophore through the transformation of a dark meso-ester-BODIPY to an emissive meso-carboxylate-BODIPY and validated its utility for light-triggered anticancer drug [92] release and fluorescence monitoring [93]. Zhu and co-workers reported a BODIPY-functionalized carboplatin-based Pt(IV) anticancer prodrug with enhanced activation and improved cytotoxicity against cancer cells [94]. Sampedro et al. studied hierarchical self-assembly of BODIPY dyes as a technique for improving capsaicin’s anticancer efficacy in prostate cancer [95].

To overcome the limitation of PDT due to its oxygen dependency, PDT in conjunction with an oxygen-independent treatment is urgently needed. Through the application of a photothermal treatment (PTT) agent to convert light into heat, PTT kills cancer cells by causing hyperthermia [96,97]. On the other hand, heat-shock proteins can also considerably minimize the damage that hyperthermia causes to cells during PTT by making malignant cells more heat-resistant [98]. Zhao et al. studied how heavy metals influenced the PDT/PTT performance of certain halogenated AzaBODIPYs, such as diiodo, dibromo, or dichloro-substituted Aza-BODIPY cores [99]. An Aza-BODIPY scaffold has been embedded with four alkyl groups (-C_8_H_17_) to improve the solubility and to uplift its capacity to self-assemble into nanoparticles in the presence of DSPE-PEG5000. The most effective PDT/PTT agent was found to be Aza-BODIPYphoto-d (Figure 6), which showed potential for imaging-guided PDT and PTT for malignancies. A single-molecule FRET (smFRET)-based photosensitizer BDP-CR, was developed by Fan et al. to accomplish NIR FLI in conjunction with combined PDT and PTT [100]. Two diiododistyrylbodipy (DIBDP) and one croconaine (CR) molecule were joined by click chemistry to produce BDP-CR, where DIBDP and CR are the PDT and PTT active agents, respectively. During normoxic conditions, the light-excited (671-nm) DIBDP created ROS via PDT, and CR produced heat for PTT through smFRET. Thus, the excited photosensitizer caused hyperthermia in hypoxic environments, which killed malignant cells by PTT. Therefore, in tumor therapy, the synergistic PDT and PTT treatments increased the therapeutic index to create “1 + 1 > 2” results.

Researchers in this field are constantly attempting to improve the architecture and potency of photosensitizers, not limited to BODIPY dimers, for PDT applications [101]. BODIPY dimers with intersystem crossing (ISC) have recently been described as triplet photosensitizers, and their potential uses in photodynamic treatment (PDT), photocatalysis, and photon upconversion are encouraging [101]. Developing BODIPY dimers entails joining two BODIPY molecules. The development of such molecules must consider variables like stable emission and absorption wavelengths and the ability to create ROS efficiently when activated by light. BODIPY dimers must have the appropriate photophysical features for PDT, such as intense absorption at the therapeutic region. Cakmak et al. recently reported orthogonal BODIPY dimers (Di-BODIPY1, Di-BODIPY2, and Di-BODIPY3 in Figure 6) [102]. The singlet oxygen quantum yields were 51, 46, and 21% for compounds Di-BODIPY1, Di-BODIPY2, and Di-BODIPY3, respectively. Cancerous cells have been employed to verify phototoxicity for the dimers. Wu et al. synthesized an orthogonal BODIPY dimer Di-BODIPY4 and two hetero-BODIPY dimers Di-BODIPY5 and Di-BODIPY6 to extend the absorption wavelength [103]. Fluorescence quantum yields of Di-BODIPY4, Di-BODIPY5, and Di-BODIPY6 were estimated to be 2.2, 17.6, and 2.3%, correspondingly. The triplet state lifetimes of Di-BODIPY4 and Di-BODIPY5 were measured to be 115.6 and 140.9 μs (using nanosecond transient absorption spectroscopy).

## 3. Summary

BODIPY scaffolds have excellent optical characteristics, chemical flexibility, and compatibility making them critical substrates for developing functional fluorescent markers for a wide range of biomedical applications. Their contributions include bioimaging, disease diagnostics, drug administration, and sensor development for monitoring a variety of biological processes. Researchers continue to investigate and innovate with BODIPY-based fluorophores, broadening their medical applications. Continued advancements in synthesis methods, structural design, and functionalization are expected to enhance their specificity and sensitivity for imaging and sensing applications. Furthermore, the integration of BODIPY into multifunctional platforms, such as theranostic agents, could usher in a new era of personalized medicine. Overcoming current challenges and exploring innovative applications will likely solidify BODIPY-based molecules as indispensable tools in the biomedical field.
